# Supplementary material for: A next-generation, histological atlas of the human brain and its application to automated brain MRI segmentation
Source: bioRxiv. 2024 Sep 6:2024.02.05.579016. Preprint. [Version 2] doi: 10.1101/2024.02.05.579016 (PMC11398399; doi:10.1101/2024.02.05.579016)
Supplement: Supplement 5 [file media-5.pdf]

Supplementary information

Supplementary Methods for

A next-generation, histological atlas of the human brain and its application to automated brain MRI segmentation

Adrià Casamitjana[1,2], Matteo Mancini[1,3,4], Eleanor Robinson[1], Loïc Peter[1], Roberto Annunziata[1], Juri Althonayan[1], Shauna Crampsie[1], Emily Blackburn[1], Benjamin Billot[1,5], Alessia Atzeni[1], Oula Puonti[6,7], Yaël Balbastre[1,7], Peter Schmidt[8], James Hughes[8], Jean C Augustinack[7], Brian L Edlow[7,9], Lilla Zöllei[7], David L Thomas[10,11], Dorit Kliemann[12], Martina Bocchetta[10,13], Catherine Strand[14], Janice L Holton[14], Zane Jaunmuktane[14], & Juan Eugenio Iglesias[1,5,7,*]

1 Department of Medical Physics and Biomedical Engineering, University College London, London, United Kingdom
2 Research Institute of Computer Vision and Robotics, University of Girona, Girona, Spain
3 Department of Cardiovascular, Endocrine-Metabolic Diseases and Aging, Italian National Institute of Health, Rome, Italy
4 Cardiff University Brain Research Imaging Centre, Cardiff University, Cardiff, United Kingdom
5 Computer Science and Artificial Intelligence Laboratory, Massachusetts Institute of Technology, Cambridge, MA, USA
6 Danish Research Centre for Magnetic Resonance, Centre for Functional and Diagnostic Imaging and Research, Copenhagen University Hospital - Amager and Hvidovre, Copenhagen, Denmark
7 Athinoula A. Martinos Center for Biomedical Imaging, Massachusetts General Hospital and Harvard Medical School, Boston, MA, USA
8 Advanced Research Computing Centre, University College London, London, United Kingdom
9 Center for Neurotechnology and Neurorecovery, Department of Neurology, Massachusetts General Hospital and Harvard Medical School, Boston, MA, USA
10 Dementia Research Centre, Department of Neurodegenerative Disease, UCL Queen Square Institute of Neurology, University College London, London, United Kingdom
11 Neuroradiological Academic Unit, Department of Brain Repair and Rehabilitation, UCL Queen Square Institute of Neurology, University College London, London, United Kingdom
12 Department of Psychological and Brain Sciences, University of Iowa, Iowa City, IA, USA
13 Centre for Cognitive and Clinical Neuroscience, Division of Psychology, Department of Life Sciences, College of Health, Medicine and Life Sciences, Brunel University London, United Kingdom, Department of Psychology, Brunel University London, London, United Kingdom
14 Queen Square Brain Bank for Neurological Disorders, Department of Clinical and Movement Neurosciences, UCL Queen Square Institute of Neurology, University College London, London, United Kingdom

* Correspondence: jiglesiasgonzalez@mgh.harvard.edu

Manual delineation of brain structures

Our neuroanatomical protocols include 333 regions of interest (ROIs): 34 cortical, and 299 subcortical. The ontology of these ROIs is largely based on the Allen reference brain [1] (henceforth “Allen atlas”). We use their protocol because, leaving aside the sparsity of annotated sections and low sample size (N=1), this atlas leads the pack in terms of number and detail of labels. Our ontology is provided as a spreadsheet in the Supplementary Data. The cortical labels follow the Desikan-Killiany atlas [2]. Our cortical anatomical protocol considers the cerebral cortex as a single ROI in the histology; the subdivision into the 34 ROIs is propagated from the *ex vivo* MRI scans, which were automatically parcellated with FreeSurfer [3]. Our subcortical protocol relies mostly on two atlases: the Allen atlas and the Mai atlas [4]. In addition to these, we used complementary atlases and protocols for specific brain regions, as detailed below; we note that the set of atlases selected to label each region was decided by our senior neuroanatomists (JCA, MB, DK) based on the visibility and clarity of ROIs in our LFB sections.

- *Hippocampus:* In addition to the Allen and Mai atlases, the hippocampal subfields were segmented following [5-8]. The layers of the hippocampus (i.e., stratum lacunosum-moleculare, stratum radiatum, stratum pyramidale, stratum oriens, stratum lucidum) were labelled according to the Allen atlas.

- *Hypothalamus:* The hypothalamus was first segmented into five subregions (anterior superior, anterior inferior, superior tuberal, inferior tuberal, and posterior) as defined in [9,10]. The nuclei within those subregions were then labelled according to [11], as well as the Allen and Mai atlases. Other guiding references were [12-14].

- *Amygdala:* The amygdala was segmented into nine subregions (lateral nucleus, basal nucleus, accessory basal nucleus, central nucleus, medial nucleus, cortical nucleus, anterior amygdaloid area, cortico-amygdaloid transition area), typically visible in *ex vivo* MRI data. The segmentation followed a combination of: *(i)* the Allen and Mai atlases; and *(ii)* a protocol we previously developed for the amygdala atlas in FreeSurfer [15], based on human histology and morphometry resources [16-19].

- *Basal ganglia:* The basal ganglia structures (caudate, putamen and the external and internal segments of the globus pallidus) were traced following the anatomical definitions provided by the Allen and Mai atlases.

- *Basal forebrain:* Structures of the basal forebrain (basal nucleus of Meynert, nucleus of diagonal band, nucleus subputaminalis, substantia innominata) were labelled according to the Allen atlas.

- *Thalamus:* Delineations for 67 thalamic regions were made by three neuroanatomists (M.B., J.A., E.R.), who discussed and agreed to a consistent protocol based on the Allen and Mai atlases, supplemented by [4,20-22] to accommodate for minor heterogeneities in thalamic anatomy – including differences in histological preparation. Following a first round of segmentations, difficult segmentations were reviewed by the more senior neuroanatomist with expertise in thalamic anatomy (M.B.).

- *Midbrain:* The most caudal part of the midbrain was cut with the brainstem, resulting in axial slices, while the cranial part was cut with the cerebrum, resulting in coronal slices. Therefore, we had to adopt two different reference segmentation criteria for the midbrain. For the coronal sections, the primary references used for labelling midbrain regions were the Allen and Mai atlases. For the axial sections, Duvernoy's Atlas of the Human Brain Stem and Cerebellum [23] and [24] were used as additional references.

- *Cerebellum:* The delineation of the molecular and granular layers of the cerebellum as well as cerebellar white matter followed the definitions from the Allen atlas and Duvernoy's Atlas of the Human Brain Stem and CerebelluM [23].

- *Brainstem:* The brainstem was labelled by three neuroanatomists (J.A., E.B., E.R.), who discussed and agreed to a consistent protocol based on the Allen atlas and Duvernoy's Atlas of the Human Brain Stem and Cerebellum [23], as well as on other sources [4,24,25], to accommodate for minor heterogeneities brainstem anatomy and differences in histological preparations.

Brain regions were delineated by our junior neuroanatomists E.R., J.A. (brainstem, midbrain, thalamus, cerebellum and E.B. (hippocampus, brainstem), with guidance and feedback from our senior neuroanatomists D.K. (amygdala), Z.K. (hippocampus, hypothalamus), and M.B. (hippocampus, hypothalamus, thalamus). We note that, by having a single senior neuroanatomist in charge of defining the protocol of each brain region, we maximise the consistency in the annotations – since the junior neuroanatomists in charge of delineating a given region were all trained by the same person.

Supplementary References

1 Ding, S. L. *et al.* Comprehensive cellular-resolution atlas of the adult human brain. *Journal of comparative neurology* **524**, 3127-3481 (2016).

2 Desikan, R. S. *et al.* An automated labeling system for subdividing the human cerebral cortex on MRI scans into gyral based regions of interest. *Neuroimage* **31**, 968-980 (2006).

3 Fischl, B. *et al.* Automatically parcellating the human cerebral cortex. *Cerebral cortex* **14**, 11-22 (2004).

4 Mai, J. K., Majtanik, M. & Paxinos, G. *Atlas of the human brain*. (Academic Press, 2015).

5 Dalton, M. A., Zeidman, P., Barry, D. N., Williams, E. & Maguire, E. A. Segmenting subregions of the human hippocampus on structural magnetic resonance image scans: An illustrated tutorial. *Brain and Neuroscience Advances* **1**, 2398212817701448 (2017).

6 de Flores, R. *et al.* Characterization of hippocampal subfields using ex vivo MRI and histology data: Lessons for in vivo segmentation. *Hippocampus* **30**, 545-564 (2020).

7 Wisse, L. *et al.* Subfields of the hippocampal formation at 7 T MRI: in vivo volumetric assessment. *Neuroimage* **61**, 1043-1049 (2012).
8 Iglesias, J. E. *et al.* A computational atlas of the hippocampal formation using ex vivo, ultra-high resolution MRI: application to adaptive segmentation of in vivo MRI. *Neuroimage* **115**, 117-137 (2015).
9 Bocchetta, M. *et al.* Detailed volumetric analysis of the hypothalamus in behavioral variant frontotemporal dementia. *Journal of Neurology* **262**, 2635-2642 (2015).
10 Makris, N. *et al.* Volumetric parcellation methodology of the human hypothalamus in neuroimaging: normative data and sex differences. *NeuroImage* **69**, 1-10 (2013).
11 Baroncini, M. *et al.* MRI atlas of the human hypothalamus. *Neuroimage* **59**, 168-180 (2012).
12 Parker, J. A. & Bloom, S. R. Hypothalamic neuropeptides and the regulation of appetite. *Neuropharmacology* **63**, 18-30 (2012).
13 Gabery, S. *et al.* Changes in key hypothalamic neuropeptide populations in Huntington disease revealed by neuropathological analyses. *Acta neuropathologica* **120**, 777-788 (2010).
14 Schindler, S. *et al.* Development and evaluation of an algorithm for the computer-assisted segmentation of the human hypothalamus on 7-Tesla magnetic resonance images. *PloS one* **8**, e66394 (2013).
15 Saygin, Z. M. *et al.* High-resolution magnetic resonance imaging reveals nuclei of the human amygdala: manual segmentation to automatic atlas. *Neuroimage* **155**, 370-382 (2017).
16 Mai, J. K. & Paxinos, G. *The human nervous system*. (Academic press, 2011).
17 Gloor, P. & Guberman, A. H. The temporal lobe & limbic system. *Canadian Medical Association. Journal* **157**, 1597 (1997).
18 Sims, K. & Williams, R. The human amygdaloid complex: a cytologic and histochemical atlas using Nissl, myelin, acetylcholinesterase and nicotinamide adenine dinucleotide phosphate diaphorase staining. *Neuroscience* **36**, 449-472 (1990).
19 Freese, J. L. & Amaral, D. G. *Neuroanatomy of the primate amygdala*. (The Guilford Press, 2009).
20 Mai, J. K. & Majtanik, M. Toward a common terminology for the thalamus. *Frontiers in Neuroanatomy* **12**, 114 (2019).
21 Ilinsky, I. *et al.* Human motor thalamus reconstructed in 3D from continuous sagittal sections with identified subcortical afferent territories. *eneuro* **5** (2018).
22 Forutan, F. *et al.* Organisation and maturation of the human thalamus as revealed by CD15. *Journal of Comparative Neurology* **437**, 476-495 (2001).
23 Naidich, T. *Duvernoy's atlas: the human brain stem and cerebellum*. (Am Soc Neuroradiology, 2007).
24 Hirsch, W. L. *et al.* Anatomy of the brainstem: correlation of in vitro MR images with histologic sections. *American journal of neuroradiology* **10**, 923-928 (1989).
25 Mtui, E., Gruener, G. & Dockery, P. *Fitzgerald's Clinical Neuroanatomy and Neuroscience E-Book*. (Elsevier Health Sciences, 2020).
